# Fractional order tracking control of a disturbed differential mobile robot

**DOI:** 10.1371/journal.pone.0321749

**Published:** 2025-05-27

**Authors:** José Ignacio Aguilar-Pérez, Manuel Armando Duarte-Mermoud, Martín Velasco-Villa, Rafael Castro-Linares

**Affiliations:** 1 Mechatronics Section, Electrical Engineering Department, Cinvestav-IPN, Mexico City 07360, Mexico; 2 Facultad de Ingeniería y Arquitectura Universidad Central de Chile Santiago, Chile; Imperial College London, UNITED KINGDOM OF GREAT BRITAIN AND NORTHERN IRELAND

## Abstract

A trajectory tracking problem for a wheeled mobile robot, under skidding and slipping effects, is addressed. A sliding mode control scheme with fractional order reaching dynamics is proposed to solve the problem. The design of the control scheme is based on backstepping and passivity feedback equivalence techniques. The skidding and slipping effects are treated as a part of exogenous disturbances associated to a kinematic model of the robot. A formal study, based on Lyapunov stability theory, is carried out to assure the existence of a sliding surface where trajectory tracking is obtained; the same stability tool is used to study the trajectory tracking error behavior of the closed loop system. The performance of the control scheme is evaluated by means of numerical simulations and experimental results showing a good performance when compared with the corresponding integer order control scheme.

## 1 Introduction

The importance of wheel mobile robots (WMR) has been increasing in recent years [1, 2] due to its role in the transport of materials and people together with the large areas of applications. Among these applications, agriculture has emerged as a prominent field where mobile robots play a crucial role in enhancing efficiency and sustainability. For instance, the development of robotic vehicles equipped with advanced functionalities, such as seed selection mechanisms, has shown promising results in automating tasks traditionally performed manually. The work presented in [3] demonstrates a significant step towards integrating autonomy in agricultural practices, emphasizing the potential of robotics to address challenges in modern farming.

This growth was initially driven by the kinematic representation of the different types of wheel-driven mobile robots followed by their respectively dynamic modeling[4]. Taking into consideration the inherent constraints due to the position of the wheels on the body of the vehicle, the most commonly used mobile robots are non-holonomic vehicles deferentially driven or unicycle type vehicles, mainly because of the simplicity in their construction. This type of robots will be considered in this work.

The study of the control of non-holonomic mobile robots gained importance since it is not possible to obtain a smooth, time-invariant feedback that would solve the problem of stabilization or trajectory tracking problems [5]. Among the most used solutions is the one proposed in [6], based on a Lyapunov’s approach, which offers a global solution to the problem; the study carried out in [7], which is based on a backstepping approach, also globally solves that problem as well.

The mobile robot control problem was extended to the case of robots affected by wheel slip and skidding disturbances described by kinematic models. A nonlinear tracking controller is presented in [8] while an adaptive tracking controller is used in [46]. A sliding mode control strategy is considered in [9] too. Also, an observer-based control strategy is considered in [10] for the robot kinematic model. In [10] it is considered an observer-based strategy. The dynamic model case has also been considered; in [11] the case of disturbance attenuation is studied and in [12] an adaptive controller is designed.

On the other hand, when trying to improve the existing strategies in both, the disturbed and disturbance-free cases, the research community has offered solutions based on standard control techniques combined with strategies based on fractional calculus in different forms. The fundamental approach has been the use of standard solutions modified by means of fractional PID control actions. Non disturbed mobile robots are studied in [13] and a PI^*a*^D^*b*^ controller is presented for the trajectory tracking problem using the robot dynamic model. In [14] a fractional PID is used to control a robot represented by its dynamic model. In [15], the same problem is studied by means of a fractional PI control action. In that research, a virtual integer control for the velocities is proposed and a fractional control strategy for the dynamic subsystem is considered. In [16], a robust strategy is proposed to deal with external disturbances through the use of a fractional order fuzzy-PID controller, while in [17] a combination of a backstepping strategy with a fractional order Fuzzy-PID controller is used. Disturbances generated by possible actuator failures are considered in [18] where a fractional order sliding mode controller is proposed. The consideration of a dynamic model in the aforementioned works simplifies the treatment of the non-holonomic constraint in a differential mobile robot since the robot is modeled as two subsystems; one of them represents the kinematic relations of the robot and the second one represents the dynamic part, as it is done in [19] where a cascade structure is considered.

The regulation problem under disturbances was addressed in [20] using fractional order sliding modes. A strategy based on fractional order PID control actions is modified in [21] where neural networks and optimization techniques are included. In [22], an integer order controller is generalized to fractional order by modifying the states in the integer feedback solution. On the other hand, in [23] a leader-follower formation problem is considered by means of a fractional order sliding mode controller and in [24] a fractional order sliding mode is also proposed to deal with the synchronization problems of mobile robots.

Some of the control strategies mentioned above often result in chattering in the control signals. In [25], a controller that reduces high-frequency vibrations in the control signal, without considering the effects of skidding and lateral slipping, is presented. On the other hand, fractional order sliding mode (FOSM) control has been developed as a solution to address this limitation, as discussed in [25–27].

In this article, a control technique based on the backstepping approach, feedback passivation, and sliding mode control with fractional order reaching dynamics (SMCFORD) is presented to address the trajectory tracking problem in the presence of uncertainties associated with skidding and lateral slipping. Specifically, a sliding mode technique with fractional order reaching dynamics is used to enhance robustness of the resultant closed loop system, assuming that skidding and lateral slipping disturbances are not known. Besides, a formal analysis, using Lyapunov stability theory, is carried out to show the attraction of the closed loop system dynamics to a sliding surface where trajectory tracking is assured. Lyapunov stability theory is also used to study the trajectory tracking error behavior of that system. Additionally, numerical simulations and experimental results are presented to evaluate the performance of the proposed controller making a comparison with respect to an integer order sliding mode controller that solves the same trajectory tracking problem,

The paper is organized as follows. In Section 2, the mathematical model of a WMR is given where the effects of skidding and lateral slipping are considered. In Section 3, basic concepts of fractional calculus and passivity feedback equivalence are given. In Section 4, the trajectory tracking problem considered and the proposed controller are presented together with a formal analysis that allows one to ensure the existence of a sliding mode; the trajectory tracking error behavior of the closed loop system is also studied. In Section 5, numerical simulations and experimental results are presented. Finally, some concluding remarks are given in Section 6.

## 2 Kinematic model with skidding and slipping effects

**Fig 1:**
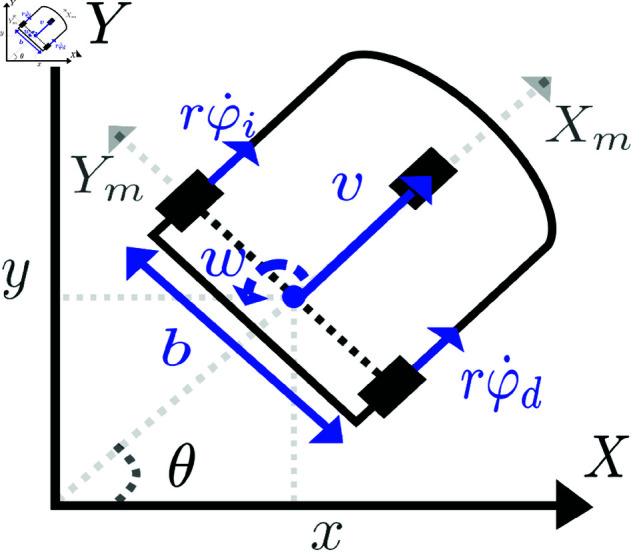
Differential drive wheeled mobile robot.

A classical kinematic model of a differential mobile robot, shown in Fig 1, can be described on the fixed *X*–*Y* plane as [4],

$$\dot{x}(t)=v(t)\cos(\theta (t))\;\dot{y}(t)=v(t)\sin(\theta (t))\;\dot{\theta }(t)=w(t)$$ (1)

where the Cartesian position (*x*,*y*) is obtained with respect to a point *p* located at the midpoint of the axles between the wheels. The orientation of the robot is given by the angle θ measured with respect to the *X*-axis. It is assumed that robot (1) moves on a flat horizontal working surface, that the wheels are non-deformable, and that the wheels rotate about an axis orthogonal to the vertical line. Model (1) satisfies the non-holonomic restriction,


$$\dot{x}(t)\sin(\theta (t))-\dot{y}(t)\cos(\theta (t))=0.$$

**Fig 2:**
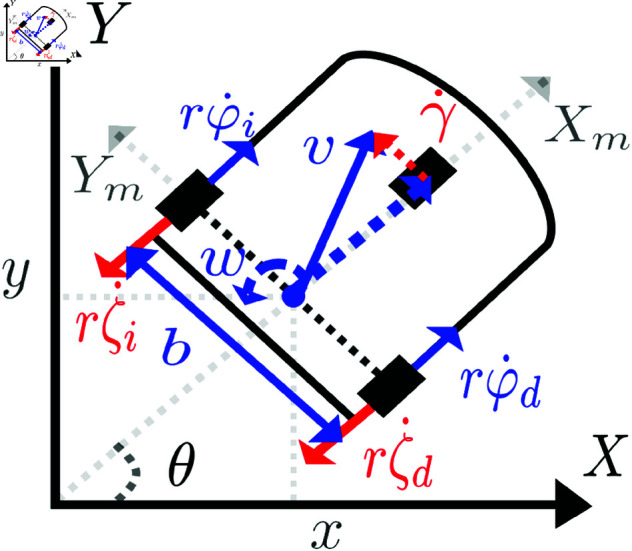
Reference frames and external disturbances in a differential WMR.

A differential mobile robot affected by skidding and slipping disturbances is shown in Fig 2, where in addition to the inertial frame *X*–*Y*, the mobile frame $X_{m}\;-\;Y_{m}$ with origin at point *p* is depicted.

In Fig 2, $\varphi_{r}$ and $\varphi_{l}$ correspond to the angular positions of the right and left wheels, respectively, while ${\dot{\varphi }}_{r}$ and ${\dot{\varphi }}_{l}$ represent their respective angular velocities. Additionally, $\zeta_{r}$ and $\zeta_{l}$ correspond to the skidding disturbances on the right and left wheels, respectively.

The lateral displacement related to the slipping disturbances is characterized by the velocity vector $\dot{\gamma }$. The parameter *r* corresponds to the wheel radius. The angular wheel velocities, and the linear velocity *v* and angular velocity *w*, are related by the following relation,

$$[\begin{array}{c} v\\ w \end{array}]=\frac{1}{2b}[\begin{array}{cc} b & b\\ 1 & -1 \end{array}][\begin{array}{c} {\dot{\varphi }}_{r}\\ {\dot{\varphi }}_{l} \end{array}].$$ (2)

The wheel restrictions on the robot of Fig 2 produce [28],

$$\dot{x}\cos(\theta )+\dot{y}sen(\theta )+b\dot{\theta }=r({\dot{\varphi }}_{r}-{\dot{\zeta }}_{l})\;\dot{x}\cos(\theta )+\dot{y}sen(\theta )-b\dot{\theta }=r({\dot{\varphi }}_{r}-{\dot{\zeta }}_{l})\;-\dot{x}sen(\theta )+\dot{y}\cos(\theta )=\dot{\gamma }.$$ (3)

Defining the linear velocity on each wheel as ${\dot{\rho }}_{r}=r({\dot{\varphi }}_{r}-{\dot{\zeta }}_{r})$, ${\dot{\rho }}_{l}=r({\dot{\varphi }}_{l}-{\dot{\zeta }}_{l})$ it is possible to define a state vector for the perturbed system in Fig 2 as,


$$\eta =[\begin{array}{cccccccc} x & y & \theta & \gamma & \rho_{r} & \rho_{l} & \varphi_{r} & \varphi_{l} \end{array}]T$$

then, it is possible to rewrite the constraint equations (3) in terms of the state variables as,


$$A(q)\dot{q}=0$$

where


$$A(q)=[\begin{array}{cccccccc} -sen(\theta ) & \cos(\theta ) & 0 & -1 & 0 & 0 & 0 & 0\\ \cos(\theta ) & sen(\theta ) & b & 0 & -1 & 0 & 0 & 0\\ \cos(\theta ) & sen(\theta ) & -b & 0 & 0 & -1 & 0 & 0 \end{array}].$$

The null space of matrix *A*(*q*), 𝒩(q) can be expanded by the columns of the matrix *S*(*q*) in such a way that,


A(q)S(q)=0

with


$$S(q)=[\begin{array}{ccccc} -sen(\theta ) & \frac{\cos(\theta )}{2} & \frac{\cos(\theta )}{2} & 0 & 0\\ \cos(\theta ) & \frac{sen(\theta )}{2} & \frac{sen(\theta )}{2} & 0 & 0\\ 0\; & \frac{1}{2b} & -\frac{1}{2b} & 0 & 0\\ 1 & 0 & 0 & 0 & 0\\ 0 & 1 & 0 & 0 & 0\\ 0 & 0 & 1 & 0 & 0\\ 0 & 0 & 0 & 1 & 0\\ 0 & 0 & 0 & 0 & 1 \end{array}].$$

Therefore, from [29] it follows that $\dot{q}$ belongs to 𝒩(q) and therefore can evolve in the form,

$$\dot{q}=S(q)\eta$$ (4)

where η takes the form,

$$\eta =[\begin{array}{ccccc} \dot{\gamma } & {\dot{\rho }}_{l} & {\dot{\rho }}_{r} & {\dot{\varphi }}_{l} & {\dot{\varphi }}_{r} \end{array}]T_{.}$$ (5)

Therefore, from equation (4), it is obtained,

$$\dot{x}=\frac{{\dot{\rho }}_{r}+{\dot{\rho }}_{l}}{2}\cos(\theta )-\dot{\gamma }sen(\theta )\;\dot{y}=\frac{{\dot{\rho }}_{r}+{\dot{\rho }}_{l}}{2}sen(\theta )+\dot{\gamma }\cos(\theta )\;\dot{\theta }=\frac{{\dot{\rho }}_{r}-{\dot{\rho }}_{l}}{2b}.$$ (6)

Considering now the relation (2) and the configuration of the disturbances on Fig 2, it is possible to get,


$$v-v_{p}=\frac{{\dot{\rho }}_{r}+{\dot{\rho }}_{l}}{2}$$

$$w-w_{p}=\frac{{\dot{\rho }}_{r}-{\dot{\rho }}_{l}}{2b}$$

where *v*,*w* are the linear and angular velocities respectively, and $v_{p},w_{p}$ are the perturbations in the linear and angular velocities respectively. Therefore, the set of equations (6) can be rewritten as,

$$\dot{x}=(v-v_{p})\cos(\theta )-\dot{\gamma }sen(\theta )\;\dot{y}=(v-v_{p})sen(\theta )+\dot{\gamma }\cos(\theta )\;\dot{\theta }=w-w_{p}.$$ (7)

**Remark 1.** *It should be pointed out that the kinematic model (*7*) shows the effects of skidding and slipping disturbances that in general cannot be measured. Under these circumstances, model (*7*) represents a starting point in order to decouple or attenuate the disturbance effects. Also, it is important to note that skidding and slipping disturbances are exogenous phenomena that appear under particular conditions, in conjunction with the friction of the surface of contact with the wheels, and depend on both linear and angular velocities. This means that the disturbances are not always present [30].*

## 3 Basic concepts of fractional calculus and passivity

In this section, some basic concepts related to fractional calculus and passivity theory are recalled. These concepts are necessary to present a solution to the trajectory tracking problem for the differential drive mobile robot (7) with skipping and slipping effects.

Fractional calculus primarily encompasses the computation of derivatives and integrals with fractional orders, a domain applicable across diverse scientific and engineering disciplines [31], as demonstrated in studies such as [32] . While there are several definitions of fractional derivatives, there is only one definition of fractional integrals; this is the Riemann-Liouville fractional integral. The Riemann-Liouville α order fractional integral of a function *f*(*t*) is defined as [33],


$$t_{0}I_{t}^{\alpha }f(t)=D_{t}^{-\alpha }f(t)=\frac{1}{\Gamma (\alpha )}\int_{t_{0}}^{t}{\left(t-\tau \right)}^{\alpha -1}f(\tau )d\tau$$

where *t*_0_ is the initial time, α>0 and *f*(*t*) is integrable. Γ(·) is the Euler gamma function defined by $\Gamma (z)=\int_{0}^{\infty }e^{-t}t^{z-1}dt$. The Euler gamma function extends the concept of exponentiation to arbitrary real (or complex) numbers, thus implicating the calculation of fractional derivatives. One common fractional derivative of a function *f*(*t*) of order α is the Riemann-Liouville fractional derivative defined as,


$${\;}_{t_{0}}^{RL}D_{t}^{\alpha }f(t)=\frac{d^{n}}{dt^{n}}\left({\;}_{t_{0}}I_{t}^{n-\alpha }f(t)\right)=\frac{1}{\Gamma (n-\alpha )}\frac{d^{n}}{dt^{n}}\int_{t_{0}}^{t}\frac{f(\tau )}{{\left(t-\tau \right)}^{\alpha -n+1}}d\tau$$

where n−1<α≤n, n∈𝐍, being **N** the set of natural numbers. Another, fractional derivative of a function *f*(*t*) of order α is the Caputo fractional derivative defined as,


$${\;}_{t_{0}}^{C}D_{t}^{\alpha }f(t)={\;}_{t_{0}}I_{t}^{n-\alpha }\left(\frac{d^{n}}{dt^{n}}f(t)\right)=\frac{1}{\Gamma (n-\alpha )}\int_{t_{0}}^{t}\frac{f^{\left(n\right)}(\tau )}{{\left(t-\tau \right)}^{\alpha -n+1}}d\tau$$

where, as above, n−1<α≤n, n∈𝐍. The previous definitions lead to denote the integration of a function *f*(*t*) when α<0 and its differentiation when α>0; when α=1 one has the first order derivative of the function [33].Some special properties of fractional calculus are summarized as follows [33–36]:

1. When α>0, the fractional derivative of *f*(*t*) is the left inverse of the fractional integral,
  $${\;}_{t_{0}}^{RL}D_{t}^{\alpha }{\;}_{t_{0}}I_{t}^{\alpha }f(t)={\;}_{t_{0}}^{C}D_{t}^{\alpha }{\;}_{t_{0}}I_{t}^{\alpha }f(t)=f(t).$$
2. The Caputo fractional derivative of a constant *C* satisfies,
  $${\;}_{t_{0}}^{C}D_{t}^{\alpha }C=0.$$
3. From the definition of the Riemann-Liouville and Caputo fractional derivatives, the following holds for α>0,
  $$\frac{d^{n}}{dt^{n}}\left({\;}_{t_{0}}^{RL}D_{t}^{\alpha }f(t)\right)={\;}_{t_{0}}^{RL}D^{n+\alpha }f(t),\;{\;}_{t_{0}}^{C}D^{\alpha }\left(\frac{d^{n}}{dt^{n}}f(t)\right)={\;}^{C}D_{t}^{\alpha }f(t)$$
  where n∈𝐍.
4. When 0<α<1, the following equality holds almost everywhere:
  $${\;}_{t_{0}}I_{t}^{\alpha }{\;}_{t_{0}}^{C}D_{t}^{\alpha }=f(t)-f(t_{0}).$$
5. If *f*(*t*) is continuous on $\left[t_{0},t_{f}\right]$ and 0<α<1, then
  $${\;}_{t_{0}}I_{t}^{\alpha }f(t_{0})=0.$$

In order to simplify the notation, $D^{\alpha }f(t)=f^{\left(\alpha \right)}(t)$ is used to denote the Riemann-Liouville fractional derivative ${\;}_{t_{0}}^{RL}D_{t}^{\alpha }f(t)$, together with the notation $I^{\alpha }f(t)=D^{-\alpha }f(t)=f^{\left(-\alpha \right)}(t)$ to represent the fractional integral ${\;}_{t_{0}}I_{t}^{\alpha }f(t)$. It is important to notice that the application of the aforementioned fractional order integral and derivative definitions is impractical, leading to the use of numerical methods to carry out these computations. For this purpose, a low-cost computational method based on [37] was used. Commonly employed techniques include those based on the Grunwald-Letnikov approach [38].

**Remark 2.** *Some advantages of fractional calculus in the design of control systems are:*

- **Enhancements in Robustness***: Fractional order controllers have demonstrated increased robustness against parameter variations within the system. This robustness stems from the ability of fractional operators to capture more complex dynamics, providing a better fit for systems with uncertainties or disturbances [39].*
- **Improvements in Convergence Speed***: The inherent smoothness of fractional derivatives allows functions, such as the saturation function sat(x), to exhibit more gradual transitions. This characteristic reduces the likelihood of oscillations or overshoots, facilitating a faster and more stable convergence to the desired equilibrium or reference point.*
- **Flexibility in Controller Design***: -1By permitting derivative orders between 0 and 1, fractional order controllers provide a broader spectrum of adjustments. This flexibility enables fine-tuning of the system’s behavior, better aligning with the specified performance requirements [40].*

Some additional important properties of fractional calculus can be found in [41]. Passivity is a concept that describes the behavior of a system or component in response to input signals. A passive system is one that dissipates, rather than generates, energy during its operation. Passivity is often employed to ensure stability and performance. A passive system will not amplify input signals; instead, it tends to absorb or dissipate energy rather than magnify it. This characteristic can be desirable in control systems to prevent the system from becoming unstable or oscillating excessively. Passivity, passivity feedback equivalence and their relation to the stabilization of nonlinear systems is discussed in detail in [42] and [43]. Let us consider a multi-input multi-output (MIMO) nonlinear system described by,

$$\dot{x}=f(x)+g(x)u\;y=h(x)$$ (8)

where $x\in {𝐑}^{n}$ is the state, $u\in {𝐑}^{m}$ is the input vector and $y\in {𝐑}^{m}$ is the output vector. The components of vector *f*(*x*), vector *h*(*x*) and those in the *m* columns of matrix *g*(*x*) are assumed to be $C^{\infty }$ (continuous differentiable) functions. It is said that system (8) is passive from the input *u* to the output *y* if there exists a $C^{\infty }$ non-negative function $V:{𝐑}^{n}\to 𝐑$ with V(0)=0, called *storage function*, such that satisfies,


$$V(x)-V(x^{0})\leq \int_{0}^{t}y^{T}(s)u(s)ds$$

where $x^{0}=x(0)\in {𝐑}^{n}$, t≥0. In [42], it is shown that if system (8) has relative degrees *r*_1_ = 1, *r*_2_ = 1,...,*r*_*m*_ = 1, it is weakly minimum phase and the matrix *L*_*g*_*h*(0) is nonsingular, then it is locally equivalent to a passive system. In fact, in the new coordinates (*y*,*z*), with *z* being a set of complementary coordinates, system (8) can be rewritten as,

$$\dot{y}=a(y,z)+b(y,z)u\;\dot{z}=f^{*}(z)+p(y,z)y+[\Sigma_{i=1}^{m}q_{i}(y,z)y_{i}]v$$ (9)

where *b*(*y*,*z*) is nonsingular for all (*y*,*z*) near (0,0). *p*(*y*,*z*) and the *q*_*i*_(*y*,*z*)’s are suitable matrices of appropriate dimensions and $\dot{z}=f^{*}(z)$ are the *zero dynamics* of the system. When substituting the feedback,


$$u=b^{-1}(y,z)[-a(y,z)+v]$$

where *v* is a new input vector, system (9) takes the form,


$$\dot{y}=v$$

$$\dot{z}=f^{*}(z)+p(y,z)y+[\Sigma_{i=1}^{m}q_{i}(y,z)y_{i}]v.$$

Since it is assumed that system (8) or, equivalently, system (9) is weakly minimum phase, its zero dynamics are Lyapunov stable with a time-independent and *C*^2^ Lyapunov function W^*^(*z*). One then chooses the input vector *v* as,

$$v=[I+M(y,z){]}^{-1}[-(L_{p(y,z)}W^{*}(z){)}^{T}+w]$$ (10)

where $M(y,z)=[(L_{q_{i}}W^{*}){\;}^{T}\cdots (L_{q_{m}}W^{*}){\;}^{T}{]}^{T}$. The choice (10) makes the closed loop system $[{\dot{y}}^{T}{\dot{z}}^{T}]=\bar{f}(y,z)+\bar{g}(y,z)w$ passive from the input *w* to the output *y*. If, in addition, this passive system is locally zero state detectable^1^, then its equilibrium (y,z)=(0,0) can be made asymptotically stable by the simple feedback w=−ϕ(y) with ϕ(0)=0 and $y^{T}\phi (y)>0$ for each y≠ 0.

## 4 The trajectory tracking problem

### 4.1 Problem statement

The position trajectory tracking errors are defined as $e_{x}=x_{t}\;-\;x$ and $e_{y}=y_{t}\;-\;y$ and the orientation trajectory tracking error is defined as $e_{\theta }=\theta_{t}\;-\;\theta$. *x*_*t*_, *y*_*t*_ and $\theta_{t}$ are the position and orientation trajectories to be tracked and are obtained from the kinematic model of an ideal differential mobile robot defined as,

$${\dot{x}}_{t}(t)=v_{t}(t)\cos(\theta_{t}(t))\;{\dot{y}}_{t}(t)=v_{t}(t)\sin(\theta_{t}(t))\;{\dot{\theta }}_{t}(t)=w_{t}(t)$$ (11)

where $v_{t}$ and *w*_*t*_ are the linear and angular velocities respectively. Since there is an offset in position and orientation between the kinematics of the perturbed model and the ideal kinematic model, a transformation of the tracking errors is carried out, this is,

$$[\begin{array}{c} x_{e}\\ y_{e}\\ \theta_{e} \end{array}]=[\begin{array}{ccc} cos(\theta ) & sin(\theta ) & 0\\ -sin(\theta ) & cos(\theta ) & 0\\ 0 & 0 & 1 \end{array}][\begin{array}{c} e_{x}\\ e_{y}\\ e_{\theta } \end{array}].$$ (12)

Taking the first derivative with respect to time of the trajectory tracking errors given by (12) and substituting the dynamics (7) and (11) leads to the following trajectory error dynamics,

$${\dot{x}}_{e}=wy_{e}+v_{t}\cos(\theta_{e})-v+g_{1}\;{\dot{y}}_{e}=-wx_{e}+v_{t}\sin(\theta_{e})+g_{2}\;{\dot{\theta }}_{e}=w_{t}-w+g_{3}$$ (13)

where

$$g_{1}=-w_{p}y_{e}+v_{p}\;g_{2}=w_{p}x_{e}-\dot{\gamma }\;g_{3}=w_{p}$$ (14)

are unknown disturbance terms, which include skidding and lateral slipping effects, and are assumed to be bounded as it is stated in the following Assumption.

**Assumption 1.** *The disturbance terms are assumed to be bounded, that is, they satisfy,*

$$|g_{1}|\leq \eta_{1},\;|g_{2}|\leq \eta_{2},\;|g_{3}|\leq \eta_{3}$$ (15)

*where* $\eta_{1}$, $\eta_{2}$ *and* $\eta_{3}$ *are non-zero positive real constants.*

**Remark 3.** *It is worth noting that the bounds $\eta_{1}$, $\eta_{2}$ and $\eta_{3}$ given in (15) are linked to practical thresholds since, as mentioned in Remark 1, the disturbances g_1_, g_2_ and g_3_ are associated to the skidding and slipping exogenous phenomena which, at the same time, depend on the linear and angular velocities of the WMR. In fact, knowledge of the bounds $\eta_{1}$, $\eta_{2}$ and $\eta_{3}$ is fundamental for the design of the proposed control scheme. In many real applications, an estimate of these bounds can be obtained from the knowledge of the WMR properties, though it may imply complicated computations. A different approach would be the design and inclusion of a disturbance estimator in the control scheme. Such an approach constitutes a future research topic.*

The trajectory tracking problem considered in this work consists in design a control scheme such that $[x_{e}(t)\;y_{e}(t)\;\theta_{e}(t){]}^{T}\to 0$ as t→∞ in the presence of the disturbance terms *g*_1_, *g*_2_ and *g*_3_ which include skidding and slipping effects.

### 4.2 Solution to the tracking problem

The approach used in this section draws inspiration from the research conducted by [46].

*Step 1*.

Let us recall the dynamics of *y*_*e*_ from (13), this is,

$${\dot{y}}_{e}=-wx_{e}+v_{t}\sin(\theta_{e})+g_{2}.$$ (16)

First, it is considered that the disturbance term *g*_2_ is not present in (16), leading to the dynamics,

$${\dot{y}}_{e}=-wx_{e}+v_{t}\sin(\theta_{e}).$$ (17)

Based on the sliding mode control technique [44], the following switching function is proposed:


$$s_{1}=y_{e}+k_{y1}\int_{0}^{t}y_{e}(\tau )d\tau$$

where *k*_*y*1_ is a positive real constant. The switching function (4.2) allows to define the commutation surface *s*_1_ = 0. On this surface it also holds that,

$${\dot{s}}_{1}={\dot{y}}_{e}+k_{y1}y_{e}=0.$$ (18)

The solution of this equation is such that $y_{e}\to 0$ when t→∞, this is *y*_*e*_ converge to 0 asymptotically (exponentially) on the surface *s*_1_ = 0. To attract the dynamics of system (17) to *s*_1_ = 0, the following fractional order reaching dynamics is chosen for *s*_1_:

$$s_{1}^{\left(\kappa_{1}+1\right)}=-k_{1}sign(s_{1})$$ (19)

with $0<\kappa_{1}\leq 1$. $k_{1}>0\in \mathbb{R}$ and *sign*(*s*_1_) being the *sign* function defined as,


$$sign(s_{1})=\left\{ \begin{array}{cc} \;\;\;1, & if\;s<0\\ -1, & if\;s>0. \end{array}\right.$$

By differentiating (19) to the order $-\kappa_{1}$, which is equivalent to integrate (19) to the order $\kappa_{1}$ [31], one obtains,


$${\dot{s}}_{1}=-k_{1}{\left(sign(s_{1})\right)}^{\left(-\kappa_{1}\right)}.$$

From (4.2) one has that ${\dot{s}}_{1}={\dot{y}}_{e}+k_{y1}y_{e}$, thus,

$${\dot{y}}_{e}+k_{y1}y_{e}=-k_{1}{\left(sign(s_{1})\right)}^{\left(-\kappa_{1}\right)}.$$ (20)

Substituting the dynamics (17) in (20), we obtain,

$$-wx_{e}+v_{t}\sin(\theta_{e})+k_{y1}y_{e}=-k_{1}{\left(sign(s_{1})\right)}^{\left(-\kappa_{1}\right)}.$$ (21)

In accordance to the backstepping technique [43], let us consider,


$$\alpha_{1}=v_{t}\sin(\theta_{e})$$

to be a virtual control input in the dynamics (17). From (21) the following expression is then obtained for $\alpha_{1}$:

$$\alpha_{1}=-k_{y1}y_{e}+wx_{e}-k_{1}{\left(sign(s_{1})\right)}^{-(\kappa_{1})}$$ (22)

which assures the attraction to *s*_1_ = 0. This can be verified when considering the Lyapunov function candidate,

$$V_{s_{1}}=\frac{1}{2}s_{1}^{2}.$$ (23)

Since $\alpha_{1}$ assures that ${\dot{s}}_{1}=-k_{1}\left(sign(s_{1})\right){\;}^{\left(-\kappa_{1}\right)}$, ${\dot{V}}_{s_{1}}$ takes the form,


$${\dot{V}}_{s_{1}}={\dot{s}}_{1}s_{1}=-k_{1}{\left(sign(s_{1})\right)}^{\left(-\kappa_{1}\right)}s_{1}.$$

Taking into account that $s_{1}=|s_{1}|sign(s_{1})$ along with $sign(s_{1})=sign(\left(sign(s_{1})\right){\;}^{\left(-\kappa_{1}\right)})$, one has that,


$${\left(sign(s_{1})\right)}^{\left(-\kappa_{1}\right)}=|{\left(sign(s_{1})\right)}^{\left(-\kappa_{1}\right)}|sign({\left(sign(s_{1})\right)}^{\left(-\kappa_{1}\right)})$$

$$=|{\left(sign(s_{1})\right)}^{\left(-\kappa_{1}\right)}|sign(s_{1}).$$

Then,


$${\dot{V}}_{s_{1}}=-|s_{1}|(k_{1}|{\left(sign(s_{1})\right)}^{\left(-\kappa_{1}\right)}|)<0$$

and attraction to the surface *s*_1_ = 0 is achieved.

*Step 2*.

Let us now consider the function,

$$y=v_{t}\sin(\theta_{e})-\alpha_{1}$$ (24)

as the output associated to system (13). From (22) and (24), the dynamics of *y*_*e*_ in (13) is written as,

$${\dot{y}}_{e}=y-k_{y1}y_{e}-k_{1}{\left(sign(s_{1})\right)}^{\left(-\kappa_{1}\right)}+g_{2}.$$ (25)

Differentiating (24) with respect to time, one has that,

$$\dot{y}={\dot{v}}_{t}\sin(\theta_{e})+v_{t}{\dot{\theta }}_{e}\cos(\theta_{e})-{\dot{\alpha }}_{1}$$ (26)

where,


$${\dot{\alpha }}_{1}=-k_{y1}\left[y-k_{y1}y_{e}-k_{1}{\left(sign(s_{1})\right)}^{\left(-\kappa_{1}\right)}+g_{2}\right]\;-k_{1}{\left(sign(s_{1})\right)}^{-(\kappa_{1}+1)}+\dot{w}x_{e}\;+w\left[wy_{e}+v_{t}\cos(\theta_{e})-v+g_{1}\right]\;.$$

By substituting (25) into (20), the dynamics (26) can be rewritten in the form,


$$\dot{y}=\alpha_{2}+wv+\Delta g$$

where,


$$\alpha_{2}=\;{\dot{v}}_{t}\sin(\theta_{e})+v_{t}(w_{t}-w)\cos(\theta_{e})\;+k_{y1}\left[y-k_{y1}y_{e}-k_{1}{\left(sign(s_{1})\right)}^{\left(-\kappa_{1}\right)}\right]\;+k_{1}{\left(sign(s_{1})\right)}^{-(\kappa_{1}+1)}\;-\dot{w}x_{e}-w\left[wy_{e}+v_{t}\cos(\theta_{e})\right]$$

and Δg is a disturbance term given by,


$$\Delta g=g_{3}v_{t}\cos(\theta_{e})-k_{y1}g_{2}-g_{1}w.$$

Then one obtains the new system,

$${\dot{y}}_{e}=y-k_{y1}y_{e}-k_{1}{\left(sign(s_{1})\right)}^{\left(-\kappa_{1}\right)}+g_{2}\;\dot{y}=\alpha_{2}+wv+\Delta g.$$ (27)

When *s*_1_ = 0, system (27) is minimal phase and has a relative degree equal to one. Thus, a feedback that makes this system passive can be proposed for the case in which $g_{1}=g_{2}=g_{3}=0$, that is without disturbance terms. The following feedback is proposed:

$$v=\frac{1}{w}\left(-\alpha_{2}+v_{l}\right)$$ (28)

where w≠0 and $v_{l}$ is a new input. Then system (27), with $g_{1}=g_{2}=g_{3}=0$, is rewritten as,

$${\dot{y}}_{e}=y-k_{y1}y_{e}-k_{1}{\left(sign(s_{1})\right)}^{\left(-\kappa_{1}\right)}\;\dot{y}=v_{l}.$$ (29)

The following storage function is considered:


$$V_{2}=V_{s_{1}}+\frac{1}{2}y^{2}$$

where $V_{s_{1}}$ is given by (23). The time derivative of $V_{2}$ is,


$${\dot{V}}_{2}=s_{1}{\dot{s}}_{1}+y\dot{y}.$$

Substituting ${\dot{s}}_{1}=-\left(sign(s_{1})\right){\;}^{\left(-\kappa_{1}\right)}$ and $\dot{y}$, from (29), into ${\dot{V}}_{2}$, one has that,


$${\dot{V}}_{2}=s_{1}\left[-k_{1}{\left(sign(s_{1})\right)}^{\left(-\kappa_{1}\right)}\right]+yv_{l}.$$

Using again the fact that $\left(sign(s_{1})\right){\;}^{\left(-\kappa_{1}\right)}=|\left(sign(s_{1})\right){\;}^{\left(-\kappa_{1}\right)}|sign(s_{1})$, it is concluded that ${\dot{V}}_{2}\leq yv_{l}$ and, therefore, the feedback (28) makes the system (27) passive from the input $v_{l}$ to the output *y*, when no disturbance terms are present.

For an ideal sliding mode on the surface *s*_1_ (this is, when *s*_1_ = 0), System (29) is also zero state observable. Thus, the feedback,

$$v_{l}=-k_{y2}y$$ (30)

where $k_{y2}>0\in \mathbb{R}$, achieves asymptotic stability of system (29), in accordance with [42].

Consider again the dynamics (27) with no disturbance terms. The following switching function is now proposed:

$$s_{2}=y-\int_{0}^{t}v_{l}(\tau )d\tau$$ (31)

which defines the sliding surface *s*_2_ = 0. On that surface, ${\dot{s}}_{2}=0$, that is,

$${\dot{s}}_{2}=\dot{y}-v_{l}=0$$ (32)

or, equivalently $\dot{y}=v_{l}$. Thus, on the surface *s*_2_ = 0, system (27), with no disturbance terms, is passive from input $v_{l}$ to output *y*. Besides, the output feedback (30) also achieves asymptotic stability. In order to have the system trajectories attracted to the surface *s*_2_ = 0, the following fractional order reaching dynamics is chosen for *s*_2_:


$$s_{2}^{\left(\kappa_{2}+1\right)}=-k_{2}sign(s_{2})$$

where $k_{2}>0\in \mathbb{R}$ and $0<\kappa_{2}\leq 1$. Then, from (32) one has that,


$$\dot{y}=v_{l}-k_{2}{\left(sign(s_{2})\right)}^{\left(-\kappa_{2}\right)}.$$

From this last expression and the dynamics of *y* in (27), with Δg=0, one obtains the control signal *v* given by,

$$v=\frac{1}{w}\left(-\alpha_{2}+v_{l}-k_{2}{\left(sign(s_{2})\right)}^{\left(-\kappa_{2}\right)}\right)$$ (33)

which is restricted to paths for which w≠0.

It is important to notice that, due to Assumption 1 and the characteristics of the virtual model (11), Δg is bounded, that is, Δg satisfies,


$$|\Delta g|\leq \eta_{\Delta g}$$

where $\eta_{\Delta g}$ is a nonzero positive real constant.

*Step 3*.

For the control of the mobile robot orientation, the dynamics ${\dot{\theta }}_{e}$, given in equation (13), is used, this is,

$${\dot{\theta }}_{e}=g_{3}+\omega_{t}-w$$ (34)

and the switching function *s*_3_ is defined as,

$$s_{3}=\theta_{e}+k_{\theta }\int_{0}^{t}\theta_{e}(\tau )d\tau$$ (35)

where $k_{\theta }>0\in \mathbb{R}$. On the sliding surface defined by *s*_3_ = 0, one has that ${\dot{s}}_{3}=0$, this is


$${\dot{s}}_{3}={\dot{\theta }}_{e}+k_{\theta }\theta_{e}=0.$$

The solution of the differential equation ${\dot{\theta }}_{e}+k_{\theta }\theta_{e}=0$ leads to the conclusion that $\theta_{e}\to 0$ when t→∞ on the sliding surface *s*_3_ = 0.

Similar to the previous reasoning for the switching functions *s*_1_ and *s*_2_, the following fractional order reaching dynamics is chosen for *s*_3_ in order to attract the system dynamics to *s*_3_ = 0 considering that the disturbance term is not present (this is, *g*_3_ = 0):

$$s_{3}^{\left(\kappa_{3}+1\right)}=-k_{3}sign(s_{3})$$ (36)

where $k_{3}>0\in \mathbb{R}$ is a real number and $0<\kappa_{3}\leq 1$. Differentiating (36) with respect to $\left(-\kappa_{3}\right)$, which is analogous to integrating (36) to the $\left(\kappa_{3}\right)$ order, yields to the expression,

$${\dot{s}}_{3}=-k_{3}{\left(sign(s_{3})\right)}^{\left(-\kappa_{3}\right)}.$$ (37)

From ${\dot{s}}_{3}={\dot{\theta }}_{e}+k_{\theta }\theta_{e}$ and (37), one has that,

$${\dot{\theta }}_{e}+k_{\theta }\theta_{e}=-k_{3}{\left(sign(s_{3})\right)}^{\left(-\kappa_{3}\right)}.$$ (38)

Substituting equation (34), with *g*_3_ = 0, into (38) leads to,


$$\omega_{t}-w+k_{\theta }\theta_{e}=-k_{3}{\left(sign(s_{2})\right)}^{\left(-\kappa_{3}\right)}$$

which results in the control signal,

$$w=\omega_{t}+k_{\theta }\theta_{e}+k_{3}{\left(sign(s_{3})\right)}^{\left(-\kappa_{3}\right)}.$$ (39)

When the disturbance terms *g*_1_, *g*_2_ and *g*_3_ are present, a sufficient condition can be given for the gains *k*_1_ *k*_2_ and *k*_3_ to ensure the convergence to the surfaces *s*_1_ = 0, *s*_2_ = 0 and *s*_3_ = 0, under Assumption 1, as stated in the following result.

**Theorem 1:** *Consider system (16), the dynamics of $\dot{y}$ in (27) and system (34), under Assumption 1, then the feedback (22), (33) and (39) ensure that the trajectories of these systems converge to the sliding surface s_1_ = 0, s_2_ = 0 and s_3_ = 0 at a finite time interval, with s_1_, s_2_ and s_3_ defined by (4.2), (31) and (35), if it is satisfied that,*

$$k_{i}|{\left(sign(s_{i})\right)}^{\left(-\kappa_{i}\right)}|>\Gamma_{i},\;i=1,\ldots ,3$$ (40)

*where $\Gamma_{1}=\eta_{2}$, $\Gamma_{2}=\eta_{\Delta g}$ and $\Gamma_{3}=\eta_{3}$.*

**Proof:** Consider the Lyapunov function candidates,


$$V_{s_{i}}=\frac{1}{2}s_{i}^{2},\;i=1,\ldots ,3$$

which are positive definite. The derivative with respect to time of each $V_{s_{i}}$ is given by,

$${\dot{V}}_{s_{i}}=s_{i}{\dot{s}}_{i},\;i=1,\ldots ,3$$ (41)

where

$${\dot{s}}_{1}={\dot{y}}_{e}+K_{y_{1}}y_{e},\;{\dot{s}}_{2}=\dot{y}-v_{l},\;{\dot{s}}_{3}={\dot{\theta }}_{e}+k_{\theta }\theta_{e}.$$ (42)

Substituting into (41)-(42) the dynamics of *y*_*e*_, *y* and $\theta_{e}$, with $g_{1}\neq 0$, $g_{2}\neq 0$ and $g_{3}\neq 0$, together with the feedback (22), (33) and (39), leads to,


$${\dot{V}}_{s_{i}}=-k_{i}{\left(sign(s_{i})\right)}^{\left(-\kappa_{i}\right)}s_{i}+s_{i}\gamma_{i},\;i=1,\ldots ,3$$

where $\gamma_{1}=g_{2}$, $\gamma_{2}=\Delta g$, $\gamma_{3}=g_{3}$. Using again the fact that $\left(sign(s_{i})\right){\;}^{\left(-\kappa_{i}\right)}=|\left(sign(s_{i})\right){\;}^{\left(-\kappa_{i}\right)}|sign(s_{i})$ and majoring, one gets,


$${\dot{V}}_{s_{i}}\leq -|s_{3}|(k_{i}|{\left(sign(s_{i})\right)}^{\left(-\kappa_{i}\right)}|-\Gamma_{i}),\;i=1,\ldots ,3.$$

Then, if condition (40) is fulfilled, ${\dot{V}}_{s_{i}}<0$, i=1,…,3 and convergence of each *s*_*i*_ to 0 is achieved. If, in addition, the following inequalities hold:


$$k_{i}|{\left(sign(s_{i})\right)}^{\left(-\kappa_{i}\right)}|-\Gamma_{i}\geq \rho_{s_{i}},\;i=1,\ldots ,3$$

with each $\rho_{s_{i}}$ being a positive real number different from zero, one has that,

$${\dot{V}}_{s_{i}}\leq -\rho_{s_{i}}|s_{i}|,\;i=1,\ldots ,3$$ (43)

and since $V_{s_{i}}=\frac{1}{2}s_{i}^{2}=\frac{1}{2}|s_{i}{|}^{2}$, |*s*_*i*_| can be expressed as $|s_{i}|=\sqrt{2V_{s_{i}}}$. Thus, (43) can be written as,

$${\dot{V}}_{s_{i}}\leq -\eta_{s_{i}}V_{s_{i}}^{\frac{1}{2}}(t),i=1,\ldots ,3$$ (44)

with $\eta_{s_{i}}=\rho_{s_{i}}\sqrt{2}$, i=1,...,3. Consider now the scalar differential equations


$${\dot{\nu }}_{s_{i}}(t)=-\eta_{s_{i}}\nu_{s_{i}}^{\frac{1}{2}}(t),\;i=1,\ldots ,3$$

with $\nu_{s_{i}}(0)=\nu_{s_{i}}^{0}$, i=1,...,3, and their solutions,


$$\nu_{s_{i}}(t)={\left(-\frac{\eta_{s_{i}}}{2}t+\sqrt{\nu_{s_{i}}^{0}}\right)}^{2},\;i=1,\ldots ,3.$$

Since each $V_{s_{i}}$ is a continuous function which is differentiable at *t* that satisfies the differential inequality (44). one has from the comparison lemma (see [45], chapter 3, section 3.4) that,


$$V_{s_{i}}(t)\leq {\left(-\frac{\eta_{s_{i}}}{2}t+\sqrt{V_{s_{i}}^{0}}\right)}^{2},i=1,\ldots ,3$$

with $V_{s_{i}}^{0}=V_{s_{i}}(0)$. Then, since each solution vanishes after some,


$$t_{s_{i}}\leq \frac{2}{\eta_{s_{i}}}\sqrt{V_{s_{i}}^{0}},i=1,\ldots ,3$$

each *s*_*i*_ vanishes as well and the sliding on each surface *s*_*i*_ = 0 starts after a finite time interval.

**Remark 4.** *The chosen fractional order reaching dynamics $s_{i}^{\left(\kappa_{i}+1\right)}=-k_{i}sign(s_{i})$, i=1,…,3 is not unique. Some other fractional order reaching dynamics have been proposed in the literature (see, for example [47] and the references therein). In order to compute the derivative of the sign(s) function, the saturation function sat(s) or the hyperbolic tangent function are commonly used; in this work the saturation function was used.*

**Remark 5.** *From the control design procedure (steps 1 through 3), it can be noticed that the sufficient conditions,*


$$k_{1}|{\left(sign(s_{1})\right)}^{\left(-\kappa_{1}\right)}|>\eta_{2}$$

$$k_{2}|{\left(sign(s_{2})\right)}^{\left(-\kappa_{2}\right)}|>\eta_{\Delta g}$$

$$k_{3}|{\left(sign(s_{3})\right)}^{\left(-\kappa_{3}\right)}|>\eta_{3}$$

*allow to obtain the attraction of the corresponding perturbed systems to the sliding surfaces s_1_ = 0, s_2_ = 0 and s_3_ = 0 in finite time. If the fractional order reaching dynamics are chosen such that they include a term $-k_{i0}s_{i}$ in ${\dot{s}}_{i}$, with k_i0_ being a non zero positive constant, this is,*


$$s_{i}^{\left(\kappa_{1}+1\right)}=-k_{i}sign(s_{i})-k_{i0}s_{i}^{\left(\kappa_{i}\right)},\;i=1,\ldots ,3$$

attraction to the sliding surfaces *s*_*i*_ = 0. i=1,…,3 is also achieved provided the following sufficient conditions:

$$k_{1}|{\left(sign(s_{1})\right)}^{\left(-\kappa_{1}\right)}|+k_{10}|s_{1}|>\eta_{2}\;k_{2}|{\left(sign(s_{2})\right)}^{\left(-\kappa_{2}\right)}|+k_{20}|s_{2}|>\eta_{\Delta g}\;k_{3}|{\left(sign(s_{3})\right)}^{\left(-\kappa_{3}\right)}|+k_{30}|s_{3}|>\eta_{3}$$ (45)

*hold. As it is mentioned in [48] , conditions (45) only show that a stronger attraction to s_i_ = 0 is achieved and that the attraction is higher for any value of s_i_ with k_i0_ different from zero. However, conditions (45) are more restrictive since they require the knowledge of |s_i_| at every time t.*

**Remark 6.** *It is important to notice that by achieving asymptotic stability of system (*29*), $y_{e}\to 0$ and y→0 as t→∞, which implies that,*


$$v_{t}\sin(\theta_{e})\approx wx_{e}-k_{1}{\left(sign(s_{1})\right)}^{\left(-\kappa_{1}\right)}.$$

If, in addition, the orientation error dynamics tends to zero rapidly, then,


$$wx_{e}\approx k_{1}{\left(sign(s_{1})\right)}^{\left(-\kappa_{1}\right)}.$$

Since $\left(sign(s_{1})\right){\;}^{\left(-\kappa_{1}\right)}$ is not defined at *s*_1_ = 0, *x*_*e*_ will oscillate around 0 for w≠0. This behavior is verified in the numerical simulations and experimental results described in sections 5 and 5.2.

**Remark 7.** *The proposed controller, based on the backstepping technique, exhibits a limitation when the variable w approaches zero, potentially causing singularities in the system. To mitigate this issue, a practical solution is to computationally impose a small positive value on w, ensuring it is never exactly zero. While this approach is effective in practice, it introduces a slight perturbation that may impact control precision.*

### 4.3 Tracking error stability analysis

Based on the control scheme developed in the previous subsections, the tracking error behavior of the closed loop system can be studied. Let $Y_{e}=[y_{e}\;\;y\;\;\theta_{e}{]}^{T}$. Then, the closed loop system dynamics obtained when the control inputs (33), (30) and (39) are substituted into the systems (27) and (34) take the form

$${\dot{Y}}_{e}=A_{e}Y_{e}*K_{dis}D_{dis}+G$$ (46)

where,

$$A_{e}=\left[\begin{array}{ccc} -k_{y_{1}} & 1 & 0\\ 0 & -k_{y_{2}} & 0\\ 0 & 0 & -k_{y_{3}} \end{array}\right],\;K_{dis}=\left[\begin{array}{ccc} k_{1} & 0 & 0\\ 0 & k_{2} & 0\\ 0 & 0 & k_{3} \end{array}\right]$$ (47)

$$D_{dis}=\left[\begin{array}{c} (sign(s_{1}{)}^{\left(-\kappa_{1}\right)}\\ (sign(s_{2}{)}^{\left(-\kappa_{2}\right)}\\ (sign(s_{3}{)}^{\left(-\kappa_{3}\right)} \end{array}\right],\;G=\left[\begin{array}{c} g_{2}\\ \Delta g\\ g_{3} \end{array}\right]\;.$$ (48)

One can state the following result.

**Theorem 2:** *Consider the closed loop system (46), together with Assumption 1. Then, the trajectories of the trajectory tracking errors y_e_, $\theta_{e}$ and the output signal y converge towards a sphere of radius as small as desired, provided that the constant coefficients $k_{y_{1}}$. $k_{y_{2}}$ and $k_{y_{3}}$ in the matrix A_e_ are chosen so that, for a sufficient number 𝒩>0, all its eigenvalues are located to the left of the line {p∈𝒞∣Re(p)≤−𝒩} in the complex plane 𝒞.*

**Proof:** Since the matrix *A*_*e*_ in (47) is Hurwitz, there exists a real symmetric positive definite matrix *P*_*e*_ which is a solution of the Lyapunov equation


$$P_{e}A_{e}+A_{e}^{T}P_{e}=-Q_{e}$$

for a given real symmetric positive definite matrix *Q*_*e*_. The following Lyapunov function candidate is then considered:


$$V_{e}=Y_{e}^{T}P_{e}Y_{e}$$

whose time derivative along the dynamics (46) is given by


$${\dot{V}}_{e}=-Y_{e}^{T}Q_{e}Y_{e}+2Y_{e}^{T}P_{e}[K_{dis}Ddis+G].$$

Notice that $Y_{e}^{T}Q_{e}Y_{e}\geq \lambda_{min}(Q_{e})\|Y_{e}{\|}^{2}$ where $\lambda_{min}(Q_{e})$ denotes the minimum eigenvalue of matrix *Q*_*e*_ and since *Q*_*e*_ is symmetric and positive definite, then $\lambda_{min}(Q_{e})$ is real and positive. Also, since $\left(sign(s_{i}){\;}^{\left(-\kappa_{i}\right)}=|\left(sign(s_{i})\right){\;}^{\left(-\kappa_{i}\right)}|sign(s_{i}\right)$, for *i*–1,2,3, and $\|K_{dis}\|<\bar{K}$, with $\bar{K}$ being a non zero positive constant, one has that $\|K_{dis}D_{dis}\|\leq \bar{K}$. Besides, from Assumption 1, $\|G\|\leq \bar{G}$, $\bar{G}$ also being a non zero positive constant. Then ${\dot{V}}_{e}$ can be majored as follows:


$${\dot{V}}_{e}\leq -\lambda_{min}(Q_{e})\|Y_{e}\|\left(\|Y_{e}\|-\frac{2\|P_{e}\|(\bar{K}+\bar{G})}{\lambda_{min}(Q_{e})}\right).$$

Then, for


$$\|Y_{e}\|>\frac{2\|P_{e}\|(\bar{K}+\bar{G})}{\lambda_{min}(Q_{e})}$$

${\dot{V}}_{e}<0$ and the dynamics *Y*_*e*_ have convergence to a ball defined as


$$S(0,\bar{K})=\left\{Y_{e}\in {\mathbb{R}}^{3}:\|Y_{e}\|<\frac{2\|P_{e}\|(\bar{K}+\bar{G})}{\lambda_{min}(Q_{e})}\right\}.$$

If


$$\|Y_{e}\|\geq \frac{2\|P_{e}\|(\bar{K}+\bar{G})}{\lambda_{min}(Q_{e})}+\rho_{0e}>0$$

where $\rho_{0e}$ is a positive real constant, one has that

$${\dot{V}}_{e}\leq -\lambda_{min}(Q_{e})\|Y_{e}\|\rho_{0e}.$$ (49)

Since $V_{e}=Y_{e}^{T}PeY_{e}$, then $\frac{V_{e}}{\left\|Y_{e}\right\|}\leq \|Y_{e}\|\|P_{e}\|\|\frac{Y_{e}}{\left\|Y_{e}\right\|}\|=\|Y_{e}\|\|P_{e}\|$, or, equivalently, $\|Y_{e}\|\geq \frac{1}{\sqrt{\left\|P_{e}\right\|}}V_{e}^{\frac{1}{2}}$, thus (49) can be reiterated as,

$${\dot{V}}_{e}\leq -\eta_{e}V_{e}^{\frac{1}{2}}$$ (50)

where $\eta_{e}=\lambda_{min}(Q_{e})\frac{1}{\sqrt{\left\|P_{e}\right\|}}$. As before, the solution of (50) is non negative and it is bounded by,


$$V_{e}(t)\leq (-\frac{\eta_{e}}{2}t+\sqrt{V_{e,0}}{)}^{2}$$

where $V_{e,0}=V_{e}(0)$. Thus, since the solution of (50) vanishes after some


$$t_{s,e}<\frac{2}{\eta_{e}}\sqrt{V_{e,0}}$$

*Y*_*e*_ enters the ball $S(0,\bar{K})$ after a finite time interval and all trajectories starting inside this ball do not leave it.

**Remark 8.** *From the proof of Theorem 2, it can be seen that the convergence to the ball $S(0,\bar{K})$ is established in terms of the quotient $\left[2\|P_{e}\|(\bar{K}+\bar{G})]/[\lambda_{min}(Q_{e})\right]$. This quotient involves the bound $\bar{G}$ which, at the same time, includes the bounds $\eta_{1}$, $\eta_{2}$ and $\eta_{3}$ given in Assumption 1. As stated in Remark 3, these bounds are related to practical thresholds.*

A common approach in the design of sliding mode controllers is the separation of the control action into a continuous and a discontinuous part, as it is done in [50]. However, the approach followed in the present work incorporates these two controllers’ characteristics into one design. This simplifies the computations particularly when the backstepping procedure is used.

The performance of the proposed control scheme was evaluated through numerical simulations and experimental tests as described in Section 5.

## 5 Numerical and experimental evaluation

The evaluation of the proposed tracking solution under the presence of external disturbances was carried out by considering numerical simulations and real-time experiments. In both cases it is considered that, in general, there are periods of time where the skidding and slipping disturbances are not present. To highlight the performance of the fractional order solution, the integer version solution is also considered for comparison purposes. Also, the the Root Mean Square Error, the Integral of Absolute Error and the Integral of Time-weighted Absolute Error are computed for the simulation and experimental cases. The integer controller considered in this evaluation takes the form,


$$v=\frac{1}{w}\left(-\alpha_{2}-s_{1}-k_{y2}y-k_{2}sign(s_{2})\right)$$

$$w=\omega_{t}+k_{\theta }\theta_{e}+k_{3}sign(s_{3})$$

where *s*_1_ and *y* are defined by (4.2), and (24), respectively. The SMCFORD is given by equations (33) and (39).

To evaluate the performance of the controller described in Section 4, a trajectory described by, $x_{t}=b\cos(kpt)\cos(pt)$, $y_{t}=b\cos(kpt)\sin(pt)$, was used, with parameters *b* = 1, *k* = 2, *p* = 0.6. The reference linear and angular velocity were obtained from $v_{t}={\dot{x}}_{t}\cos(\theta_{t})\;+\;{\dot{y}}_{t}\sin(\theta_{t})$ and $w_{t}=\frac{\;{\dot{x}}_{t}{\ddot{y}}_{t}\;-\;{\dot{y}}_{t}{\ddot{x}}_{t}}{v_{t}^{2}}$. Additionally, the initial conditions are set as $x_{e}(0)=0\;[m]$, $y_{e}(0)=0.8\;[m]$ and $\theta_{e}(0)=1.57\;[rad]$ for both the numerical case and real-time experiments. The implementation of SMCFORD was made in Python using the algorithm presented in [37] and can be found in (see S1 Code) . In all the figures, the label *int* is used for the integer case and *fra* for the fractional case. The label *des* is used to indicate the desired trajectory.

### 5.1 Numerical simulations

The computational simulations were carried out on a machine with the specifications detailed in Table 1.

**Table 1:** Hardware specifications of the computer used for simulations.

Specification	Details
Device	LENOVO LEGION, 81HE
Processor	Intel(R) Core(TM) i7-8750H @ 2.20GHz
RAM	16.0 GB
System Type	64-bit OS, x64-based processor

**Fig 3:**
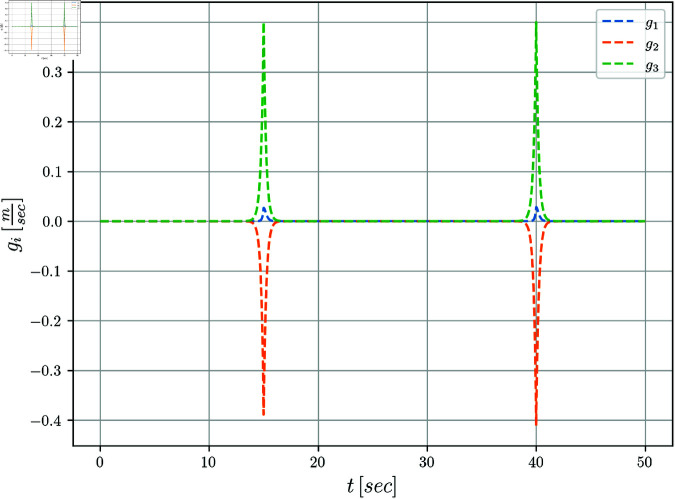
Disturbances *g*_1_(*t*), *g*_2_(*t*) **and** *g*_3_(*t*) **with respect to time.**

The kinematic model described by Equation (7) is considered. The controllers’ parameters used in the simulations are given in Table 2. The perturbations described by Equation (14) were introduced into the simulation during two time periods, as it is shown in Table 3. The dynamics of these perturbations are illustrated in Fig 3.

**Table 2:** Parameters used for integer order and fractional order controllers.

Parameter	Integer order	Fractional order
*k*_*y*1_	2	2
*k*_*y*2_	2	2
$k_{\theta }$	0.3	0.3
*k*_1_	0.01	0.01
*k*_2_	0.01	0.01
*k*_3_	0.02	0.02
$L_{1},L_{2},L_{3}$	1	–
$\kappa_{1},\kappa_{2},\kappa_{3}$	–	0.8

**Table 3:** Perturbations applied in different periods.

Period	perturbation equation	Parameters
$t_{2}\leq 20\;[sec]$	$v_{p}=k_{v_{p}}e^{-a_{v_{p}}|t-15|}$	$k_{v_{p}}=0.4,a_{v_{p}}=4.48$
	$w_{p}=k_{w_{p}}e^{-a_{w_{p}}|t-15|}$	$k_{w_{p}}=0.4,a_{w_{p}}=4.48$
	$\dot{\gamma }=k_{\dot{\gamma }}e^{-a_{\dot{\gamma }}|t-15|}$	$k_{\dot{\gamma }}=0.4,a_{\dot{\gamma }}=4.48$
$t_{1}\geq 20\;[sec]$	$v_{p}=k_{v_{p}}e^{-a_{v_{p}}|t-40|}$	$k_{v_{p}}=0.4,a_{v_{p}}=4.48$
	$w_{p}=k_{w_{p}}e^{-a_{w_{p}}|t-40|}$	$k_{w_{p}}=0.4,a_{w_{p}}=4.48$
	$\dot{\gamma }=k_{\dot{\gamma }}e^{-a_{\dot{\gamma }}|t-40|}$	$k_{\dot{\gamma }}=0.4,a_{\dot{\gamma }}=4.48$

**Fig 4:**
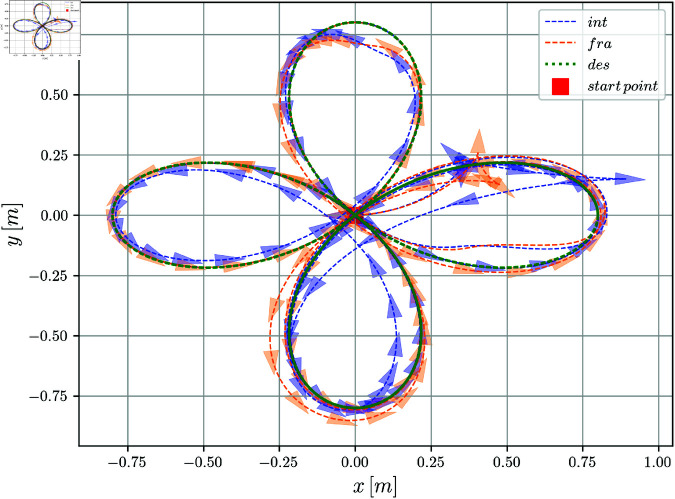
X–Y position evolution of the mobile robot.

The evolution of the robot in the *X*–*Y* plane is shown in Fig 4; the disturbances are not present throughout the entire duration of the simulation due to the characteristics of the skidding and slipping phenomena. An acceptable result was achieved, due to the convergence of the desired path for the integer and fractional order solutions. However, a better convergence was obtained for the fractional order controller.

**Fig 5:**
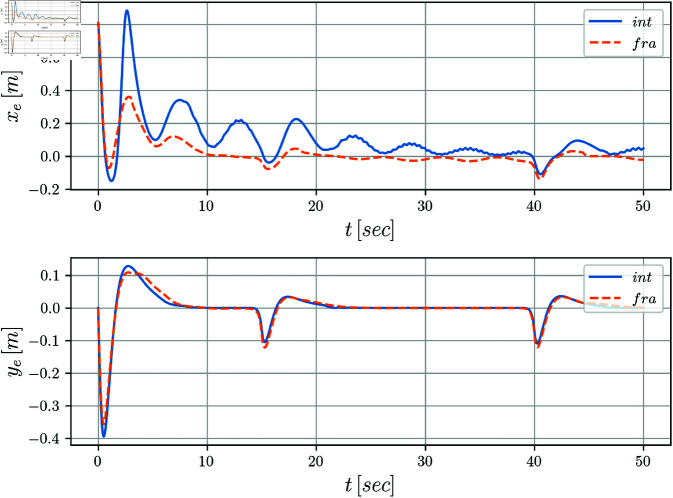
Tracking errors *x*_*e*_(*t*) and *y*_*e*_(*t*) with respect to time.

**Fig 6:**
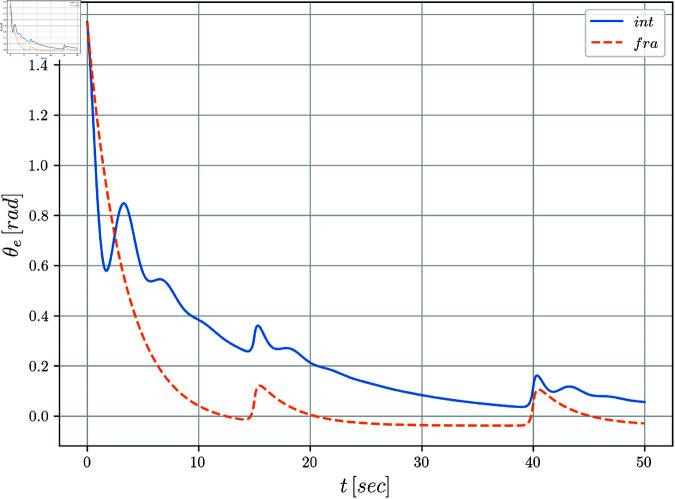
Orientation error $\theta_{e}(t)$ with respect to time.

Figs 5 and 6 display the evolution of the error coordinates $\xi_{e}=[x_{e},y_{e},\theta_{e}{]}^{T}$ over time. It can be notice a clear convergence to the origin for the tracking errors *y*_*e*_(*t*) and $\theta_{e}(t)$ when perturbations are absent in the system. The position error *x*_*e*_(*t*) seems to be more affected along the simulation. Notice that when comparing the integer versus the fractional cases, it is clear from the evolution of the tracking errors, that the fractional case improves the results obtained by the integer solution. This fact is even more evident for the periods of time when the disturbances are introduced.

**Fig 7:**
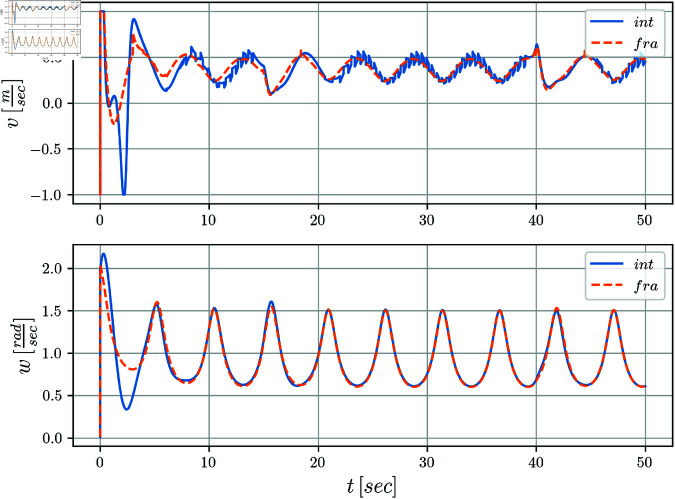
Control signals evolution v(t) and w(t) with respect to time.

Fig 7 illustrates the control signals *v*(*t*) and *w*(*t*) where the linear velocity exhibits a smooth evolution when compared to the integer case that is affected by high frequency components. No considerable distinction is observed in the signal *w*(*t*).

### 5.2 Real-time experimental results

**Fig 8:**
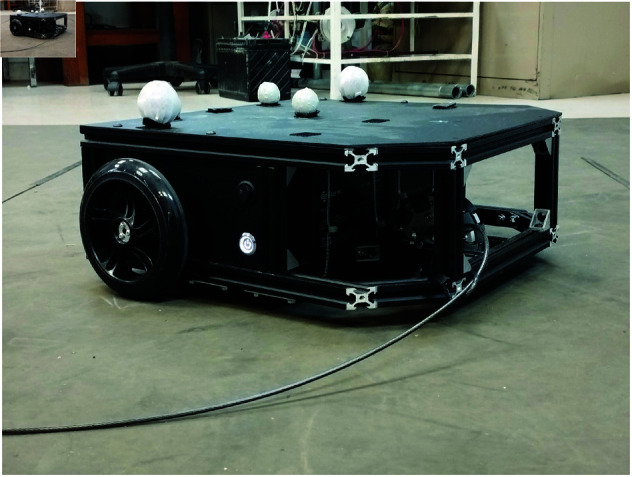
Experimental differential robot.

**Fig 9:**
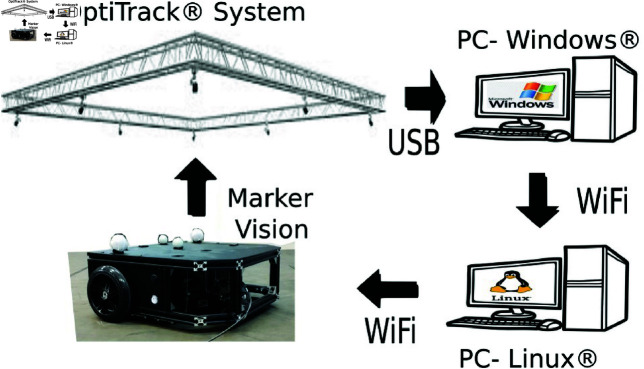
Experimental platform.

To carry out the experiments, it was considered a differential drive, non-holonomic mobile robot designed in our laboratory with two DC gearmotors of 530*rpm* and a 19:1 gear ratio that produces a torque of 9.5*kg*–*cm* with wheels of 14*cm* diameter; this gives the vehicle a velocity of 2.13*m*/*s*. The robot is equipped with a Rasberry Pi 4 to manage the robot signals and communicate with the main control PC through ROS (Robot Operating System). The robot is depicted in Fig 8. The master program that is used to link the control signals with the robot was developed in Python. Additionally, this master program connects to an OptiTrack localization system that determines the robot’s position and orientation in the working space. Fig 9 illustrates the general structure of the experimental platform. The global Optitrack localization system consists of a set of twelve Flex 3 cameras, interconnected with Motive software, from NaturalPoint Company.

In the experiments, external disturbances were introduced manually by stopping the robot to generate slippage of the wheels and applying a lateral force to cause lateral drift for approximately one second at t=0,sec and t=20,sec. The controllers’ parameters for the experiment were set as shown in Table 4.

**Table 4:** Parameters used for integer order and fractional order controllers.

Parameter	Integer order	Fractional order
*k*_*y*1_	1.9	5
*k*_*y*2_	0.5	1.1
$k_{\theta }$	2	3
*k*_1_	0.6	0.2
*k*_2_	0.1	0.2
*k*_3_	0.01	0.01
$L_{1},L_{2},L_{3}$	1	-
$\kappa_{1},\kappa_{2},\kappa_{3}$	-	0.6

It should be pointed out that the experiments were carried out involving also the natural disturbance associated with the non-punctual contact of the wheels with the working surface and the non-modeled friction forces resulting from this fact. Notice also that centrifugal and Coriolis forces, not considered in the kinematic model, are also present in the experiment.

**Fig 10:**
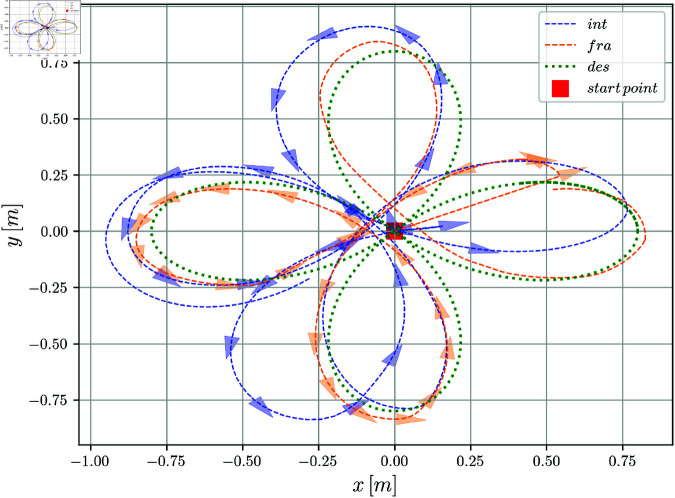
X–Y position evolution of the mobile robot.

**Fig 11:**
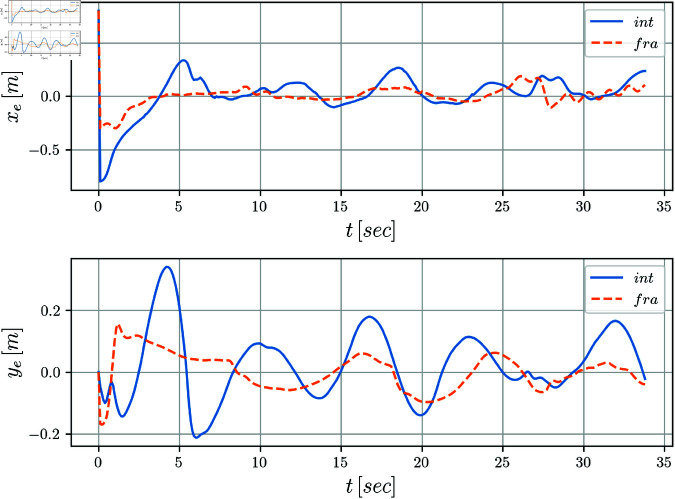
Tracking errors $x_{e}(t),\;y_{e}(t)$ with respect to time.

**Fig 12:**
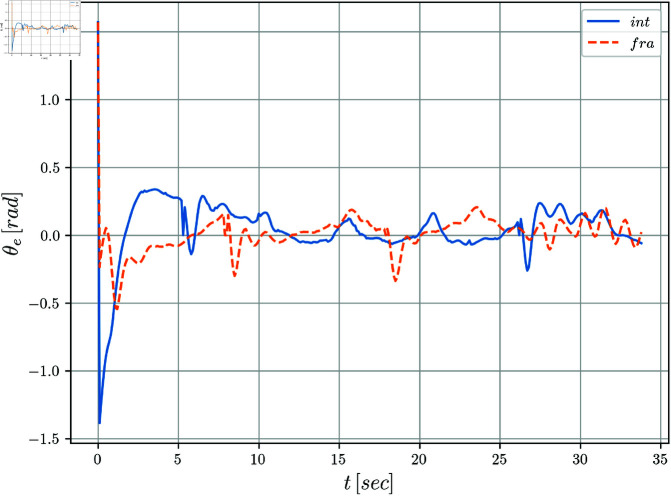
Tracking error $\theta_{e}(t)$ with respect to time.

**Fig 13:**
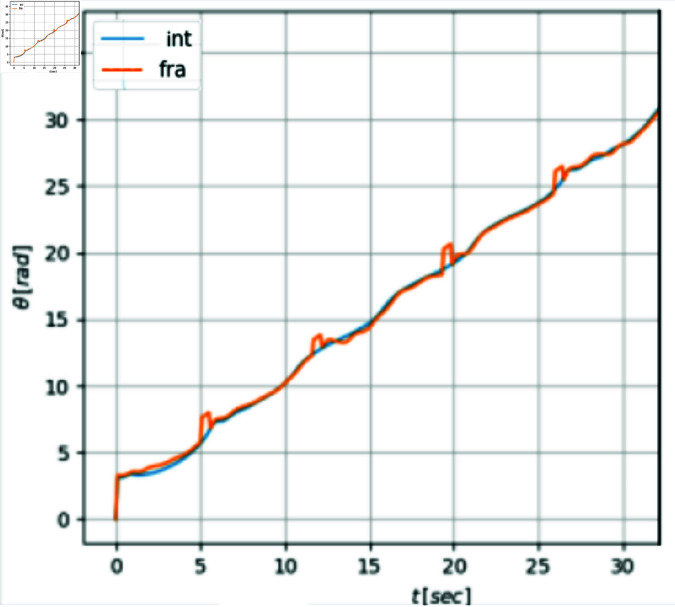
Orientation θ(t) with respect to time.

The evolution of the mobile robot in the *X*–*Y* plane is shown in Fig 10. Fig 11 shows the position tracking errors *x*_*e*_(*t*) and *y*_*e*_(*t*) while the tracking orientation error $\theta_{e}(t)$ is shown in Fig 12 and orientation with respect to time is shown in Fig 13. From these figures, it can be observed that those variables present a bounded evolution around zero for both the integer order and the fractional order controllers. In particular, the bounds obtained with the fractional order controller are smaller than the bounds obtained with the integer order controller.

**Fig 14:**
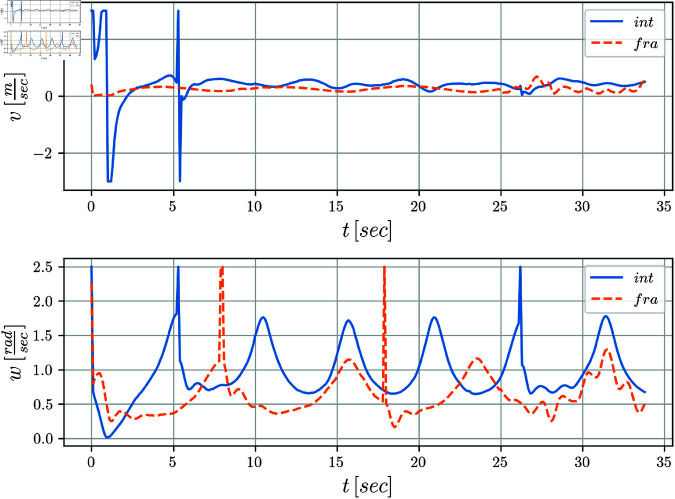
Control signals v(t),w(t) with respect to time.

Finally, in Fig 14 the control signals *v*(*t*) and *w*(*t*) are shown. The zigzag pattern in *v*(*t*) is evident in the integer case, whereas in the fractional case, a significant attenuation of this effect is observed. The signal *w*(*t*), is similar in both cases.

Since the experimental evaluation was conducted under laboratory conditions, which, while ideal, are representative of controlled environments such as factories or warehouses where mobile robots transport goods from point A to point B, it is important to consider that factors such as the weight of the transported objects can introduce unmodeled dynamics, including interactions between the wheels and the ground, which may affect the system’s stability. These experimental results indicate that, despite the model’s simplifications, the theoretical limits remain useful and applicable in real-world scenarios.

### 5.3 Root mean square error evaluation

To quantitatively compare the simulation results obtained from the integer order and the fractional order methods, the Root Mean Square Error (*RMSE*), the Integral of Absolute Error (*IAE*) and the Integral of Time-weighted Absolute Error (*ITAE*), were computed as,


$$\vartheta_{i}^{RMSE}=\sqrt{\frac{1}{n}\sum_{i=1}^{n}i_{e}^{2}(t)}$$

$$\vartheta_{i}^{IAE}=\int_{0}^{\infty }|i_{e}(t)|\;dt$$

$$\vartheta_{i}^{ITAE}=\int_{0}^{\infty }t\;|i_{e}(t)|\;dt$$

where i=x,y,θ in order to consider tracking errors position *x*_*e*_, *y*_*e*_ and its respective orientation $\theta_{e}$. The results are presented in Table 5, with each column showing these error measures for four different simulations with the same initial conditions, and each row showing the outcome of each variable for the different fractional order and integer order schemes.

**Table 5:** Error measures simulation results for integer and fractional cases.

Variable	Integer	Fractional 0.5	Fractional 0.1	Fractional 0.8
$\vartheta_{x}^{RMSE}[m]$	0.178	0.092	0.093	0.092
$\vartheta_{y}^{RMSE}[m]$	0.057	0.055	0.054	0.055
$\vartheta_{\theta }^{RMSE}[rad]$	0.346	0.281	0.280	0.283
$\vartheta_{x}^{IAE}[m]$	550.782	222.924	241.233	218.454
$\vartheta_{y}^{IAE}[m]$	113.678	122.707	120.105	123.011
$\vartheta_{\theta }^{IAE}[rad]$	1228.107	600.629	627.297	598.267
$\vartheta_{x}^{ITAE}[m]$	7746.644	2931.746	3683.994	2676.508
$\vartheta_{y}^{ITAE}[m]$	1341.152	1616.771	1634.003	1607.260
$\vartheta_{\theta }^{ITAE}[rad]$	16359.045	5213.325	6460.548	4784.704

The error measures for the experimental results are presented in Table 6. It should be pointed out, that in the experimental case, it is not possible to introduce the same disturbance in the experiment due to fact that its magnitude is non measurable.

**Table 6:** Error measures experimental results for the integer and the fractional order case.

Variable	Integer	Fractional 0.5
$\vartheta_{x}^{RMSE}[m]$	0.185	0.093
$\vartheta_{y}^{RMSE}[m]$	0.115	0.059
$\vartheta_{\theta }^{RMSE}[rad]$	0.523	0.597
$\vartheta_{x}^{IAE}[m]$	41.091	19.578
$\vartheta_{x}^{IAE}[m]$	30.467	16.526
$\vartheta_{\theta }^{IAE}[rad]$	58.565	48.676
$\vartheta_{x}^{ITAE}[m]$	5051.993	2920.521
$\vartheta_{x}^{ITAE}[m]$	4345.098	2230.385
$\vartheta_{\theta }^{IAE}[rad]$	6963.663	6354.135

From Tables 5 and 6, one can observe that the fractional order approach provides better results in terms of error measures. The ITAE metric shows a considerable reduction when using fractional orders, with the best result obtained for the 0.8 fractional controller (2676.508), representing an improvement of approximately 65.45% compared to the integer order case (7746.644).

From the experimental results presented, it is worth noting that the fractional order controller exhibits less oscillation when reaching the reference trajectory and also achieves a shorter settling time. In contrast, higher initial overshoots and more oscillations are observed when the integer order controller is used. Faster settling times could be obtained using an approach similar to the one presented in [49] where a fractional order sliding mode surface and a new combined reaching law are proposed for the trajectory tracking problem in a four-wheeled omnidirectional mobile robot.

Furthermore, it should be noted that, in the experimental results, disturbances are not only exogenous but also stem from modeling issues inherent to any kinematic model.

## 6 Conclusions

The trajectory tracking control problem for a non-holonomic wheeled mobile robot, where skidding and lateral slipping effects appear, is studied. In order to deal with such a problem, a kinematic model of the robot was obtained where the skidding and slipping effects are considered to be part of unknown bounded exogenous disturbances. The conducted experiments have shown that the system converges correctly even without explicit knowledge of the estimated limits, suggesting that the experiment has not exceeded the theoretical bound. The problem is analyzed by means of a novel sliding mode controller with fractional order reaching dynamics that was developed using the backstepping technique and passivity feedback equivalence. Using Lyapunov stability theory, sufficient conditions were given so that the existence of a sliding mode, where trajectory tracking is achieved, is ensured. These conditions involve the controller gains, the fractional order of the controller and the bounds of the unknown exogenous disturbances. Besides, it is formally shown that the sliding mode is attained within a finite time interval, The performance of the controller was evaluated by means of numerical simulations and real time experiments comparing the fractional solution with its corresponding integer solution. When computing the Root Mean Square Error (*RMSE*), the Integral of Absolute Error (*IAE*) and the Integral of Time-weighted Absolute Error (*ITAE*), it was observed that the fractional order controller exhibits acceptable performance even when considering a high velocity desired trajectory; it also achieves robustness without the need of an exact knowledge of the disturbances affecting the system. The transient performance of the controlled mobile robot could be improved by using a fractional order sliding mode surface together with a combined reaching law, as mentioned above. The incorporation of adaptive mechanisms in order to have estimates of the control actions’ parameters, as it is done in [50], could be a valuable extension of the proposal presented here since it would enable an exploration of how these techniques can complement and enhance the results obtained. An efficient planning of the desired trajectory could also be implemented. These issues and the consideration of the dynamic model of the robot are points that deserve further analysis.

## Footnotes

^1^ A system $\dot{x}=f(x)\;+\;g(x)u$, y=h(x) is locally zero state detectable if there exists a neighborhood *U* of 0 such that, for all x∈U, y(t)=h(x(t)=0 implies that x(t)→0 as t→∞. It is said to be locally zero state observable if there exists a neighborhood *U* of 0 such that, for all x∈U, y(t)=h(x(t)=0 implies that x(t)=0.
